# Pyramiding *Pita*, *Pigm*, *Pi2*, and *Xa23* to Develop Hybrid Rice with Dual Resistance to Rice Blast and Bacterial Blight

**DOI:** 10.3390/plants15020323

**Published:** 2026-01-21

**Authors:** Siyuan Wu, Xuemei Qin, Jiali Liu, Ju Gao, Lijun Gao, Geng Zhou, Yang Zhou, Tianqi Bai, Chonglie Ma, Fang Liu

**Affiliations:** 1 State Key Laboratory for Conservation and Utilization of Subtropical Agro-Bioresources, College of Agricultural, Guangxi University, Nanning 530004, China; wusiyuan610302@163.com (S.W.); qinxm2020@163.com (X.Q.); 15767163036@163.com (J.L.); 19940599676@163.com (Y.Z.); 2 Guangxi Crop Genetic Improvement and Biotechnology Laboratory, Guangxi Academy of Agricultural Sciences, Nanning 530007, China; gaojufly@126.com (J.G.); gaolijun@gxaas.net (L.G.); 15180964570@163.com (G.Z.); 3 College of Agronomy, Northwest A&F University, Yangling 712100, China; 2020013169@nwafu.edu.cn

**Keywords:** dual resistance, gene pyramiding breeding, hybrid rice varieties, *Magnaporthe oryzae*, *Xanthomonas oryzae* pv. *oryzae*

## Abstract

Rice blast and bacterial blight cause severe harm to rice production, and the breeding of resistant varieties guarantees the safety of rice production. Meanwhile, multigene pyramiding breeding based on molecular marker-assisted selection is a crucial approach for rice breeding to combat multiple diseases. This study aimed to develop accurate and efficient PARMS markers for rice blast resistance genes *Pita*, *Pigm*, and *Pi2*, and bacterial blight resistance gene *Xa23*. A systematic genotyping analysis of the resistant alleles of these 4 genes was performed on 384 major cultivated varieties in production. The results showed that only 5.21% of the varieties harbored more than two resistant alleles simultaneously. Using traditional breeding strategies in combination with the developed PARMS markers, the high-quality three-line male sterile line Ruanfeng A (pyramiding *Pita* and *Pigm*) and the strong restorer line Gui 610 (pyramiding *Pi2* and *Xa23*) were bred. Crossing these lines produced a new hybrid rice variety, Ruanfengyou 610. Ruanfengyou 610 pyramids 4 resistance genes (*Pita*/*Pigm*/*Pi2*/*Xa23*), exhibits resistance to both rice blast and bacterial blight, has prominent heterosis and excellent grain quality, and has strong application potential, which is of great significance for ensuring the safety of rice production.

## 1. Introduction

During its growth cycle, rice is vulnerable to various diseases that compromise high and stable yields as well as grain quality, posing a threat to food security at both regional and national levels. At present, pesticide application remains the primary method for managing rice diseases in production. However, this practice not only increases production costs and causes environmental harm but also exacerbates the development of drug resistance in pathogenic bacteria. Production practices have demonstrated that long-term cultivation of crops with a single resistance trait drives adaptive changes in the population structure of pathogenic physiological races. This leads to the rapid proliferation of specific dominant races, resulting in the loss of the original disease resistance in crops and a subsequent reduction in their commercial value. Rice blast, a fungal disease caused by *Magnaporthe oryzae* (an ascomycete), is one of the most devastating diseases impacting rice production. This disease can reduce rice yields by 40–50% in severe infestations and result in total crop failure in extreme cases [1,2]. Bacterial blight of rice is a disease caused by X*anthomonas oryzae* pv. *oryzae* (*Xoo*), a Gram-negative bacterium. Characterized by strong mutability, rapid transmission, and wide distribution, it is one of the three major rice diseases, posing a serious threat to the safe production of rice [3]. Frequent extreme weather in recent years has triggered recurrent outbreaks of rice blast and bacterial blight, forcing individual rice varieties to confer resistance to both diseases simultaneously. Breeding and deploying multiresistant rice cultivars is the most cost-effective and environmentally sustainable strategy. Thus, pyramiding multiple broad-spectrum, durable resistance genes to develop high-quality hybrid rice varieties with multi-disease resistance is highly essential [4]. The identification and cloning of numerous rice blast and bacterial blight resistance genes have provided key genetic resources for breeding resistant rice cultivars.

More than 140 rice blast resistance genes have been reported and designated globally, most of which are dominant genes, with only a handful being recessive genes [5]. These genes are unevenly distributed across the 12 rice chromosomes, forming distinct resistance gene clusters, with chromosomes 6, 11, and 12 being the distribution hotspots [6,7,8]. For instance, more than 10 allelic resistance genes are clustered at the *Pi2*/*9* locus on chromosome 6 [9]. Also, the *Pi1* locus on chromosome 11 contains multiple functionally related genes [10]. Similarly, at least nine rice blast resistance–specific genes have been mapped to the *R*-gene cluster in the centromeric region of rice chromosome 12 [11,12]. This clustered distribution characteristic offers crucial clues for gene cloning and evolutionary analysis. More than 30 rice blast resistance genes have been successfully cloned [13,14], among which 2 dominant genes, *Pigm* and *Pita*, have gained considerable attention due to their ability to confer durable and broad-spectrum resistance to panicle blast in rice [15,16]. The *Pigm* gene, located on chromosome 6, is closely linked or allelic to the known resistance genes *Pi2* and *Pi9* [17]. Its unique advantage lies in maintaining long-term resistance to *M. oryzae* without compromising rice yield [18,19,20], rendering it highly valuable for breeding rice varieties with durable and broad-spectrum blast resistance [21]. The encoded product of the *Pita* gene can interact with the effector protein encoded by the avirulence gene *AVR*-*Pita* of rice blast fungus to trigger disease resistance responses [22]. Also, its molecular markers have been applied in marker-assisted rice breeding [23]. *Pigm* activates disease resistance by regulating the salicylic acid signaling pathway, whereas *Pita* directly recognizes pathogen effector proteins to initiate immune defense; their synergistic action can significantly extend the durability of resistance [23].

More than 40 bacterial blight resistance genes have been identified in rice [24,25], and 17 genes, including *Xa1* [26], *Xa2* [27], *Xa3*/*Xa26* [28], *Xa7* [29], *xa13* [30], *Xa14* [27], *Xa21* [31], *Xa23* [32], *xa25* [33], *Xa27* [34], and *Xa41* [35], have been successfully cloned [36]. Among these, *Xa1*, *Xa7*, *Xa21*, and *Xa23* have been widely applied in rice breeding [27]. These genes are unevenly distributed across the rice genome, found on 10 of the 12 chromosomes (absent from chromosomes 9 and 10), with a high degree of clustering on chromosome 11 (accounting for nearly one-third of the total). Also, a relatively dense distribution is observed on chromosomes 4 and 6. At present, the use of bacterial blight resistance genes in major commercially cultivated rice varieties is low, with most applications primarily relying on single genes [37]. Among these, *Xa23* has been relatively widely applied due to its high resistance, complete dominance, and full-growth-period disease resistance [38,39]. Derived from common wild rice (*Oryza rufipogon*) in China, *Xa23* exhibits high resistance to all existing bacterial blight differential strains worldwide, including Philippine races 1–10, Chinese pathogenic races 1–7, and Japanese races 1–3. It is currently the known major bacterial blight resistance gene with the broadest resistance spectrum and strongest resistance. It also exhibits complete dominance and provides resistance throughout the entire growth period. Therefore, establishing a rice bacterial blight resistance breeding system based on the broad-spectrum resistance gene *Xa23* is crucial for producing disease-resistant rice varieties. In the three-line hybrid breeding system including cytoplasmic male sterile lines (female parents), maintainer lines, and restorer lines (male parents), integrating resistance (R) genes into restorer lines is essential for enhancing the genetic resistance of hybrid progenies [40].

The penta-primer amplification refractory mutation system (PARMS) is a high-throughput, low-cost, and automated genotyping assay based on competitive allele-specific PCR (AS-PCR) combined with a homogeneous fluorescence reporting system, specifically designed for single-nucleotide polymorphism (SNP) detection. Characterized by flexibility in SNP and sample throughput, this technique only requires standard laboratory equipment (liquid handling systems, thermal cyclers, and plate readers) and is compatible with DNA samples from diverse sources and extraction methods (e.g., alkaline lysis), making it ideal for direct PCR-based SNP marker-assisted selection (D-MAS) with advantages of simplicity, cost- and labor-saving, and robustness. Its core principle relies on a five-primer system (a pair of universal fluorescent primers, a pair of allele-specific primers, and a shared reverse primer) targeting SNPs or short indels; universal fluorescent primers are integrated into the primer design, enabling amplification of different genotypes to produce distinct fluorescent signals. Notably, the assay involves a single PCR without subsequent electrophoresis, and amplification data can be directly acquired from the original plate using a fluorescence scanner. It can significantly improve breeding efficiency and accuracy, provide strong technical support for the cultivation of superior crop varieties [41,42]. The PARMS markers have been successfully employed for the improvement of rice plant architecture and grain quality [42,43]. Using conventional breeding methods, this study developed precise and efficient PARMS functional molecular markers for marker-assisted selection. The broad-spectrum rice blast resistance genes *Pita* and *Pigm* were pyramided to create a new high-quality rice male sterile line, Ruanfeng A, with rice blast resistance. Meanwhile, the broad-spectrum rice blast resistance gene *Pi2* and the broad-spectrum bacterial blight resistance gene *Xa23* were pyramided to develop a new rice restorer line, Gui 610, with dual resistance to rice blast and bacterial blight. Crossing Ruanfeng A with Gui 610 resulted in a novel hybrid rice variety, Ruanfengyou 610. This variety has excellent grain quality and high yield and pyramids four resistance genes (*Pita*/*Pigm*/*Pi2*/*Xa23*), conferring dual resistance to rice blast and bacterial blight. It exhibits considerable commercial value and is worthy of further promotion.

## 2. Results

### 2.1. Molecular Detection of Resistance Alleles Pita, Pigm, Pi2, and Xa23 in Rice Germplasm Resources

**Figure 1:**
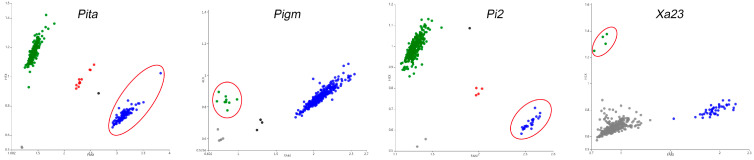
Development and genotyping of PARMS markers for *Pita*, *Pigm*, *Pi2*, and *Xa23* genes in rice germplasm resources (red circles for resistance allelic materials). FAM fluorescence signals (blue dots) and HEX fluorescence signals (green dots) corresponded to two distinct allelic types. A red dot in the fluorescence scanning result indicated that the locus was in a heterozygous state in the material. Gray dots represented negative controls or materials with locus deletions, whereas black dots denoted undetermined signals.

This study developed fluorescent molecular markers for the resistance genes *Pita*, *Pigm*, *Pi2*, and *Xa23* using the PARMS technology (Table S1). All these markers could well match the corresponding SNPs to generate fluorescence signals, thereby enabling effective and accurate analysis of the resistance genes (Figure 1). Genotyping was performed on 384 major cultivated rice varieties (Table S2). Previous studies have shown that [32], besides the variation caused by an 8 bp small fragment insertion, the susceptible allele *xa23* also has an allelic variation due to a 7.6 kb transposon insertion. Both variations can lead to the deletion of the TALE-binding element (EBE) of AvrXa23, resulting in disease susceptibility as well. The *Xa23* PARMS marker we developed could not detect the target sequence due to the presence of this large fragment insertion, resulting in no fluorescent signal. However, this marker can accurately genotype the resistant allele *Xa23* in all cases.

Among the 384 major cultivated rice varieties, 114 accessions (accounting for 29.69%) harbored the homozygous resistance allele of *Pita*, 9 accessions (2.34%) harbored *Pigm*, 25 accessions (6.51%) harbored *Pi2*, and only 4 accessions (1.04%) harbored *Xa23*. Further, 20 accessions (5.21%) harbored 2 homozygous resistance genes, whereas merely 1 accession (0.26%) carried 3 homozygous resistance genes (Table S2). The results indicate that these resistance genes have not been widely applied in rice production, and the breeding utilization of multiresistance gene pyramiding is even more limited. We performed resistance identification on some materials harbored different allelic genotypes and analyzed their resistance effects on resistance to rice blast and bacterial blight. The results showed that materials harbored single resistance alleles (*Pita*, *Pigm*, or *Pi2*) exhibited a certain level of resistance to rice blast, while the combination of *Pita*/*Pi2* or *Pita*/*Pigm* further enhanced the resistance level (Figure S2). Similarly, materials harbored the resistance allele *Xa23* demonstrated significant resistance to bacterial blight (Figure S3).

### 2.2. Breeding of High-Quality Rice Male Sterile Line Ruanfeng A by Pyramiding Pita and Pigm

Previous studies have reported that *Pita* and *Pigm* can jointly suppress pathogen expansion through various mechanisms (e.g., the broad-spectrum resistance of *Pigm* and the durable resistance of *Pita*) [17,22]. In this study, Yuanfeng B (Pita-carrying line) was first crossed with Bo B, and the resulting F_1_ progeny were further crossed with Gufeng B (*Pigm*-carrying line) to obtain double-cross F_1_ individuals. Following self-pollination, PARMS markers were used to identify plants with pyramiding homozygous *Pigm* and *Pita* genes. Two rounds of backcrossing were conducted with Bo B as the recurrent parent. Starting from the BC_2_F_2_ generation, selection was carried out by combining molecular marker screening with agronomic trait evaluation. After nine generations of backcrossing and self-pollination, the genetically stable male sterile line Ruanfeng A and its maintainer line Ruanfeng B were successfully developed (Figure S1).

**Figure 2:**
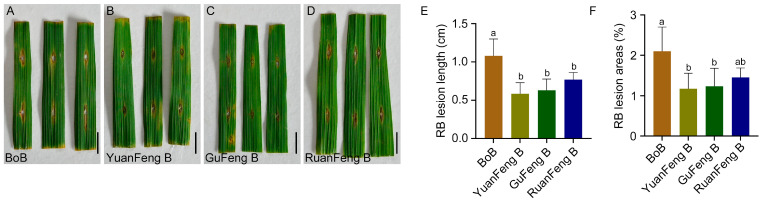
Breeding and blast resistance identification of the male sterile line Ruanfeng A/B. (**A**–**D**) Phenotypic results of BoB, YuanFeng B, GuFeng B, and RuanFeng B at 7 days post-inoculation with rice blast pathogen. Scale bar = 1.0 cm. (**E**,**F**) Lesion length (**E**) and percentage of lesion leaf area (**F**) after rice blast inoculation. Data are presented as mean ± standard deviation (*n* = 6). Different letters indicate significant differences (*p* < 0.05, one-way analysis of variance with least significant difference test).

Seedling-stage resistance identification showed that the donor parent Yuanfeng B carrying the *Pita* gene and the donor parent Gufeng B carrying the *Pigm* gene had significantly smaller lesion lengths and areas than the recurrent parent Bo B when inoculated with rice blast pathogen during the growth period. At 14 days post-inoculation with rice blast strain *CX*, the average leaf lesion area of Yuanfeng B and Gufeng B was 1.18% and 1.23%, respectively, exhibiting a resistant phenotype (Figure 2A–C,E,F and Table S3). The male sterile line Ruanfeng A and its maintainer line Ruanfeng B (pyramiding *Pigm* and *Pita*), which were bred through successive generations of backcrossing and self-pollination, also showed resistance to rice blast. These results indicated that the introduction of *Pigm* and *Pita* genes improved the rice blast resistance in Ruanfeng A and its maintainer line Ruanfeng B (Figure 2D–F and Table S3).

### 2.3. Breeding of Restorer Line Gui 610 with Dual Resistance to Bacterial Blight and Rice Blast by Pyramiding Pi2 and Xa23

**Figure 3:**
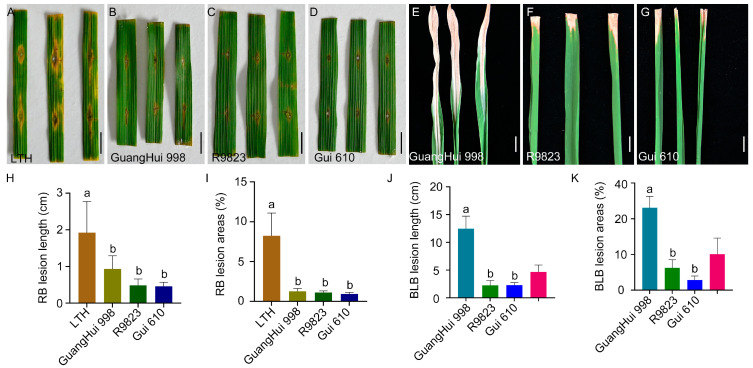
Resistance identification of restorer line Gui 610. (**A**–**D**) Phenotypic results of LTH, GuangHui 998, R9823, and Gui 610 at 7 days post-inoculation with rice blast pathogen. Scale bar = 1.0 cm. (**E**–**G**) Phenotypic results of GuangHui 998, R9823, and Gui 610 at 14 days post-inoculation with bacterial blight pathogen. Scale bar = 1.0 cm. (**H**,**I**) Rice blast lesion length (**H**) and percentage of lesion leaf area (**I**). Data are presented as mean ± standard deviation (*n* = 6). Different letters indicate significant differences (*p* < 0.05, one-way analysis of variance (ANOVA) with least significant difference (LSD) test). (**J**,**K**) Bacterial blight lesion length (**J**) and percentage of lesion leaf area (**K**). Data are presented as mean ± standard deviation (*n* = 6). Different letters indicate significant differences (*p* < 0.05, one-way ANOVA with LSD test).

Based on the genotyping results of PARMS markers, the restorer intermediate material R9823 harbored the dual resistance alleles *Xa23*/*Pi2*. The results of seedling-stage resistance identification showed that the susceptible control variety LTH exhibited an average lesion length of 1.92 cm and an average lesion coverage of 8.24% of the leaf area after inoculation with the *M. oryzae* strain *CX* (Figure 3A,H,I and Table S4). In contrast, R9823 had an average blast lesion length of only 0.482 cm and a lesion coverage of 1.10%, displaying stronger resistance than Guanghui 998, which had corresponding values of 0.93 cm and 1.26%. Furthermore, after inoculation with the *X. oryzae* pv. oryzae strain *PXO99^A^*, R9823 showed an average lesion length of 2.26 cm and a lesion areas coverage of 12.51%, which were significantly different from those of Guanghui 998 (12.46 cm in lesion length and 46.19% in leaf area coverage) (Figure 3C,F,H–K and Table S5). This significant difference preliminarily confirmed the disease-resistant effects of *Xa23* and *Pi2* genes.

Therefore, we adopted a marker-assisted breeding strategy, using Guanghui 998 as the male parent and R9823 as the female parent for cross-breeding. During the progeny selection process, the research team conducted assisted selection using *Xa23*/*Pi2* PARMS markers throughout the entire process. After eight generations of pedigree selection and continuous resistance identification, the restorer line Gui 610 was finally obtained (Figure S1). Gui 610 exhibited stable moderate resistance: after inoculation with rice blast and bacterial blight pathogens, the lesion length was 0.46 cm and 2.30 cm, the lesion areas coverage of 0.93% and 5.66%, respectively (Figure 3D,G,H–K and Tables S4 and S5). Therefore, the introduction of *Xa23* and *Pi2* genes improved the dual resistance of restorer line Gui 610 to rice blast and bacterial blight, providing new germplasm resources for rice disease-resistant breeding.

### 2.4. Breeding of Hybrid Rice Variety Ruanfengyou 610 with Dual Resistance to Rice Blast and Bacterial Blight

**Figure 4:**
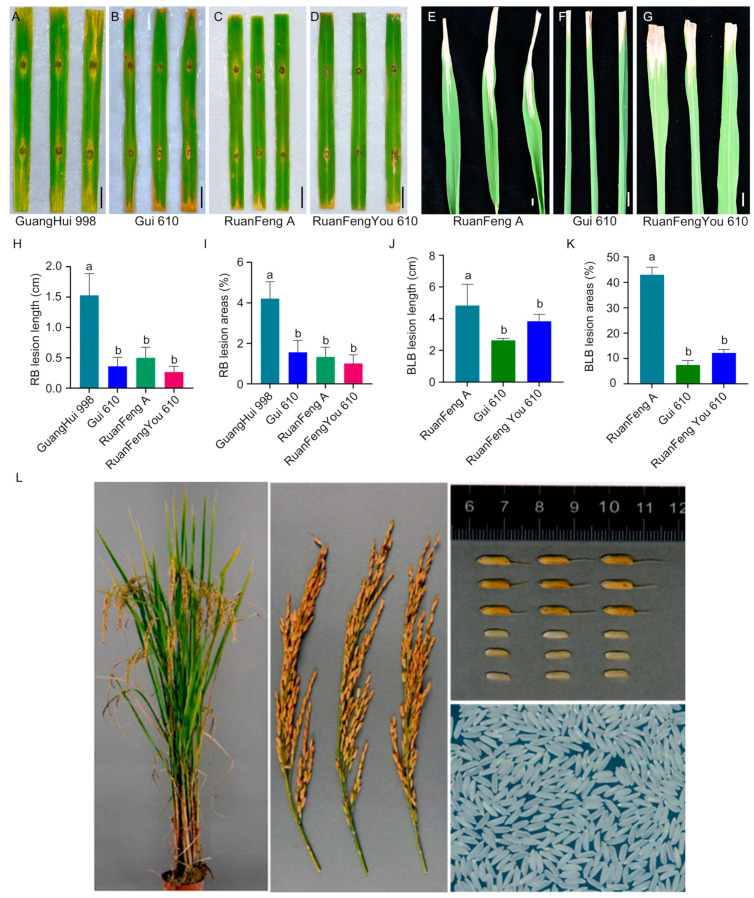
Resistance identification and agronomic phenotypic characteristics of Ruanyoufeng 610. (**A**–**D**) Phenotypic results of Ruanyoufeng 610 and its parents at 7 days post-inoculation with rice blast pathogen. Scale bar = 1.0 cm. (**E**–**G**) Phenotypic results of Ruanyoufeng 610 and its parents at 14 days post-inoculation with bacterial blight pathogen. Scale bar = 1.0 cm. (**H**,**I**) Rice blast lesion length (**H**) and percentage of lesion leaf area (**I**). Data are presented as mean ± standard deviation (*n* = 6). Different letters indicate significant differences (*p* < 0.05, one-way analysis of variance (ANOVA) with least significant difference (LSD) test). (**J**,**K**) Bacterial blight lesion length (**J**) and percentage of lesion leaf area (**K**). Data are presented as mean ± standard deviation (*n* = 6). Different letters indicate significant differences (*p* < 0.05, one-way ANOVA with LSD test). (**L**) Phenotypic characteristics of Ruanfengyou 610 on whole plant, rice panicle, grain and brown rice, and milled rice.

A test cross between Ruanfeng A and Gui 610 was conducted to develop rice varieties resistant to rice blast and bacterial blight. This cross resulted in the new hybrid rice variety Ruanfengyou 610, which pyramids four alleles (*Pita*/*Pigm*/*Pi2*/*Xa23*), successfully achieving dual resistance to rice blast and bacterial blight (Figure 4). After pyramiding the three rice blast resistance alleles (*Pita*/*Pigm*/*Pi2*), Ruanfengyou 610 exhibited a higher level of resistance to rice blast than its parental lines (Figure 4A–D,H,I). Additionally, the introgression of the resistance allele *Xa23* significantly enhanced the resistance of Ruanfengyou 610 to bacterial blight (Figure 4E–G,K,L).

After two consecutive years of regional trials, Ruanfengyou 610 was approved by the Crop Variety Approval Committee of Guangxi Zhuang Autonomous Region. Ruanfengyou 610 has a uniform population, an excellent plant and leaf architecture, a high seed-setting rate, good late-stage leaf color turning, and obvious heterosis. The plant height is 116.5 cm. When planted as late rice in southern Guangxi, its whole growth period lasts 119.2 days, with an average yield of 506.50–551.30 kg per mu, which is 5.3–7.63% higher than that of the control variety Fengtianyou 553. It is suitable for cultivation as single-cropping late rice in southwestern Guangxi. The analysis of the main grain quality indicators showed that Ruanfengyou 610 had a brown rice rate of 80.5%, a head rice rate of 58.7%, a length–width ratio of 3.3, a chalky rice rate of 6%, a chalkiness degree of 0.9%, a transparency grade of 2, an alkali spreading value of 5.2, a gel consistency of 78 mm, and an amylose content of 18.5%, meeting the high-quality Grade 3 standard specified in *NY/T593-2013 Edible Rice Variety Quality* (Table S6). In the two consecutive years of variety resistance identification, the comprehensive index of rice blast resistance was 4.0 and 4.0, respectively, with the highest panicle blast loss rate of Grade 3. The resistance was evaluated as moderately resistant. The resistance to bacterial blight was Grade 3 and Grade 5, respectively, with the resistance evaluated as moderately resistant to moderately susceptible (Table S6). It is evident that Ruanfengyou 610 is a new hybrid rice variety integrating multiple important and excellent agronomic traits such as high yield, good quality, and disease resistance (Figure 4L).

## 3. Discussion

Traditional rice disease control mainly relies on chemical pesticides. However, this method has drawbacks such as pesticide residues, environmental pollution, and increased pathogen resistance. In contrast, breeding disease-resistant varieties is widely recognized as the safest and most efficient control measure. At present, rice disease-resistant breeding has two key issues: first, the application scope of resistance genes is limited, and the coverage rate of disease resistance genes in main cultivated varieties is low; second, the utilization form of resistance genes is single, mostly based on single-gene introduction, resulting in narrow resistance spectra of varieties and easy loss of resistance due to pathogen variation. Gene pyramiding is a core strategy to break through these limitations [44]. Integrating multiple disease resistance genes with different resistance mechanisms into the same variety can significantly broaden the resistance spectrum and improve the durability of resistance [4]. Rice blast and bacterial blight are two key diseases threatening the safety of rice production in China. The academic community has carried out extensive research on the excavation and application of their resistance genes, thereby laying a foundation for disease-resistant breeding.

At present, the application of rice blast and bacterial blight resistance genes in major cultivated varieties has significant shortcomings: the number of varieties harboring effective resistance genes is small, most of which adopt single-gene application. In addition, the application rate of broad-spectrum resistance genes is extremely low. Genotypic analysis of 384 major cultivated rice germplasm resources revealed that the breeding utilization rates of rice blast resistance genes *Pita*, *Pigm*, and *Pi2* and the bacterial blight resistance gene *Xa23* were generally low in these varieties. For rice blast resistance genes, the single-gene utilization of *Pita* was dominant, whereas the pyramiding utilization of multiple genes was extremely scarce. The proportion of the broad-spectrum resistance gene *Pigm* in major cultivated varieties was less than 3%. Only four germplasm resources (accounting for 1.04%) harbored the *Xa23* resistance allele, indicating that the utilization of *Xa23* in rice breeding for bacterial blight resistance needs to be further strengthened. In terms of gene pyramiding utilization, 20 accessions (5.21%) had pyramided two homozygous resistance genes, and only 1 accession (0.26%) carried three homozygous resistance genes (Figure 1 and Table S2). These application shortcomings lead to narrow resistance spectra of varieties and easy loss of resistance due to the variation in pathogen races. Therefore, pyramiding breeding of multiple resistance genes needs vigorous development.

Rice blast resistance genes *Pita*, *Pigm*, and *Pi2* and the bacterial blight resistance gene *Xa23* are all dominant resistance genes [15,16,17,32]. Although *Pigm* and *Pi2* are alleles at the same locus [17], they can still exhibit resistance effects simultaneously in the heterozygous state. These genes can maintain their resistance characteristics in the heterozygous state of the F_1_ generation and thus can be utilized in breeding hybrid rice for resistance to rice blast and bacterial blight. In this study, PARMS molecular markers for targeting genes were developed and applied in marker-assisted selection for breeding the rice blast–resistant male sterile line Ruanfeng A by pyramiding *Pita* and *Pigm* alleles (Figure 2), and for developing the restorer line Gui 610 with dual resistance to rice blast and bacterial blight by pyramiding *Pi2* and *Xa23* alleles (Figure 3). By crossing Ruanfeng A with Gui 610, four disease resistance genes (*Pita*, *Pigm*, *Pi2*, and *Xa23*) were successfully pyramided, resulting in the development of a new high-quality hybrid rice variety Ruanfengyou 610 (Figure 4). This variety not only has dual resistance to rice blast and bacterial blight but also maintains excellent grain quality traits, thereby achieving synergistic improvement of “disease resistance and grain quality.”

Meanwhile, genotyping of 384 major cultivated rice varieties using PARMS markers revealed that the distribution frequencies of resistance genes, such as *Pita*, *Pigm*, *Pi2*, and *Xa23*, in currently commercialized varieties were extremely low, and most of them existed as single genes independently. This further confirmed the urgency of multigene pyramiding breeding. The research strategy adopted in this study breaks through the limitations of traditional single-gene breeding, which is characterized by a narrow disease-resistant spectrum and high vulnerability to pathogen adaptation. It endows rice varieties with broad-spectrum and durable disease resistance, representing a pivotal innovation in advancing breeding technology from single-trait improvement to multi-trait synergistic optimization. The marker system and gene pyramiding strategy established in this study can be directly applied to the resistance improvement of existing main cultivated varieties, thus providing a feasible approach for the rapid cultivation of multiresistant rice varieties. This gene pyramiding breeding model is replicable, target resistance genes can be replaced or added according to different disease types. It provides a reference technical framework for molecular breeding of disease-resistant rice, breaks the bottleneck of conventional rice breeding featuring single functionality and limited adaptability, and expands the application boundaries and industrialization value of rice varieties.

## 4. Materials and Methods

### 4.1. Materials

A total of 384 major cultivated rice varieties were used for developing molecular markers for target genes (Table S2). Among these, Yuanfeng B (donor of *Pita* resistance allele), Gufeng B (donor of *Pigm* resistance allele), and restorer line intermediate material R9823 (carrying dual resistance alleles *Xa23*/*Pi2*) served as disease-resistant materials, whereas the rice variety Lijiangxintuanheigu (LTH) was used as the susceptible control for rice blast. All rice materials were planted in the experimental field of Guangxi Academy of Agricultural Sciences or in greenhouses under regular water and fertilizer management. The main reagent used was 2× PARMS master mix. The main instruments included a polymerase chain reaction (PCR) instrument (Model TP600, Takara, Kyoto, Japan) and a multifunctional microplate reader (Model Infinite F200, Tecan, Männedorf, Switzerland).

### 4.2. Population Construction and Breeding Selection Process

The improvement of the Ruanfengyou 610 variety was achieved via marker-assisted backcross breeding, as illustrated in Figure S1.

F_1_ progeny were obtained by crossing Yuanfeng B with Bo B, which were then crossed with Gufeng B to construct a three-way cross F_1_ population. After self-pollination, F_2_ individuals carrying homozygous *Pigm* and *Pita* dual resistance genes were screened using PARMS-based marker-assisted selection. Two rounds of backcrossing were performed with Bo B as the recurrent parent. Starting from the BC_2_F_2_ generation, superior individuals were selected by combining molecular marker detection and agronomic trait evaluation, followed by crossing with Bo A. After nine consecutive generations of backcrossing and breeding, the male sterile line Ruanfeng A with stable agronomic traits and qualified sterility, along with its maintainer line Ruanfeng B (BC_2_F_12_), was finally obtained at the BC_9_F_1_ generation. Blast resistance identification showed a moderate resistance level. For the male parent, Guanghui 998 was crossed with R9823. Using PARMS markers to track *Xa23* and *Pi2*, a high-quality restorer line Gui 610, with moderate resistance to both rice blast and bacterial blight, was bred through eight generations of pedigree selection. Ruanfengyou 610 was officially registered and released by crossing Gui 610 with Ruanfeng A.

### 4.3. Design of Fluorescent Functional Molecular Markers, PCR Amplification, and Genotyping

Based on the reported sequence differences at the functional loci of alleles of rice blast resistance genes *Pita*, *Pigm*, and *Pi2* and bacterial blight resistance gene *Xa23*, fluorescent functional molecular markers were designed using the PARMS method [41] (Table S1).

Genomic DNA of rice was extracted from rice leaves following the CTAB method described by Murray et al. [45]. The extracted DNA was dissolved in 1× TE buffer and used as the template for subsequent PCR amplification. PCR amplification of *Pita*, *Pigm*, *Pi2*, and *Xa23* genes was performed on rice materials, with two specific forward primers and a reverse primer added simultaneously to the PCR system. The total volume of the PCR reaction system was 10 μL, comprising 5 μL of 2× PARMS master mix (containing two universal fluorescent primers, PCR buffer, dNTPs, Taq polymerase, internal reference ROX, etc.), 0.15 μL of each of the two specific-forward primers (10 mM), 0.4 μL of the reverse primer (10 mM), 1 μL of template DNA, and 3.3 μL of ddH_2_O. The PCR reaction program was as follows: initial denaturation at 95 °C for 5 min, followed by 10 touchdown cycles of 95 °C for 20 s and 65 °C (decreasing by 0.8 °C per cycle) for 1 min, and then 32 cycles of 95 °C for 20 s and 57 °C for 1 min.

The PCR products were rapidly detected using a microplate reader equipped with three fluorescence detection channels (FAM, HEX, and ROX), and the fluorescence intensity signal values were recorded. Subsequently, the fluorescence signals were analyzed via the SNP decoder 2.0 software (http://www.snpway.com:8339/, accessed on 16 December 2025) to obtain the FAM and HEX fluorescence signal intensities of each sample amplification, with each signal point output in a graphical format. Finally, genotyping was performed automatically based on fluorescence signal intensities to generate genotyping results. According to the analysis of fluorescence signal values, samples showing FAM fluorescence signals (blue dots) and HEX fluorescence signals (green dots) corresponded to two distinct allelic types. A red dot in the fluorescence scanning result indicated that the locus was in a heterozygous state in the material. Gray dots represented negative controls or materials with locus deletions, whereas black dots denoted undetermined signals.

### 4.4. Identification of Rice Blast Resistance

The detached leaf inoculation method or the spray inoculation method was employed for assessing rice blast resistance in the seedling stage. When rice plants reached the three-leaf and one-heart stage, the upper-middle portion of the penultimate leaf was selected and cut into 6 cm long leaf segments. Subsequently, a mechanical injury was created at the center of the leaf’s adaxial surface using a sterile pipette tip. A 5-μL aliquot of *M. oryzae* spore suspension (5 × 10^5^ spores/mL) was applied to the injured site to ensure uniform coverage of the wound with spores. The treated leaves were placed adaxial side up in acrylic molds, and a 0.001% 6-benzylaminopurine solution was added. The inoculated leaves were incubated in a high-humidity environment at 28 °C, with dark incubation for the first 24 h to simulate the optimal infection conditions for the pathogen. Afterward, they were transferred to a culture room at the same temperature for continued moisturized incubation, and the experimental results were recorded for 5 days post-inoculation. The *M. oryzae* strain *CX* used in this study was collected from the rice blast nursery in Limu Town, Cenxi City, Guangxi, followed by isolation and preservation. For each variety, five seedlings at the 3–4-leaf stage were selected, and the spore suspension of *M*. *oryzae* was inoculated via either syringe needle pricking on the leaf surface or direct spraying. The lesion length and area were measured at 7–10 days post-inoculation using ImageJ (Version 1.53t, National Institutes of Health, Bethesda, MD, USA) software [46].

Field identification of disease resistance in rice varieties was conducted following the industry standard of the Ministry of Agriculture of the People’s Republic of China, *Technical Regulations for Identification and Evaluation of Rice Blast Resistance in Rice Variety Trials* (NY/T 2646-2014). Natural inoculation was used to evaluate seedling leaf blast and panicle blast in rice under disease-endemic conditions. Throughout the identification process, identification nurseries were established in Limu Town, Cenci City, Guangxi (altitude: 162 m), Hezhou Institute of Agricultural Sciences in Xindu Town, Hezhou City, Guangxi (altitude: 58 m), and Yuexu Town, Jingxi City, Guangxi (altitude: 562 m). When the susceptible control reached a fully susceptible state (Grade 9), the incidence of leaf blast in the tested materials was observed, and the resistance grade was evaluated based on the severity of leaf blast.

### 4.5. Identification of Rice Bacterial Blight

The *X. oryzae* pv. *oryzae* (*PXO99^A^*) strain used in this study was obtained from the College of Agriculture, Guangxi University, and stored in a refrigerator at –80 °C. According to the method reported in previous studies [46], the *PXO99^A^* strain was inoculated on nutrient broth medium to prepare the inoculum suspension. In the rice booting stage, three plants of each rice variety were selected for inoculation using the leaf-clipping method, with three to five fully expanded leaves inoculated per plant. The lesion length and area were measured 2 weeks after inoculation using ImageJ software, and the lesions were photographed and documented using a digital camera (Nikon, Tokyo, Japan). Resistance grading based on lesion length was performed referring to the *Technical Specification for Identification of Bacterial Blight Resistance in Rice* of China (DB34/T 2810-2017). The natural incidence identification experiment of bacterial blight was conducted in the Bacterial Blight Resistance Identification Nursery for Rice Regional Trial Varieties (Combinations) of Guangxi, located in Linfeng Town, Tiandong County, Baise City, Guangxi.

### 4.6. Evaluation of Agronomic and Quality Traits

The trait in this study was evaluated according to the national agricultural industry standards of China. The determination of agronomic traits was performed referring to *Technical Specification for Regional Trials of Rice Varieties* (NY/T 1300-2007), with sampling and investigation conducted in the maturation stage. The specific method was as follows: after removing the border rows of the experimental plot, 10 representative plants were randomly selected to measure the following indicators: plant-type-related traits (plant height, stem diameter, and morphological characteristics of flag and penultimate leaves); panicle traits (number of effective panicles, panicle length, and grain number per main panicle); and yield components (1000-grain weight). Rice quality analysis was conducted according to *Quality of Edible Rice Varieties* (NY/T 593-2021), with the main detection items including appearance quality (grain size, chalkiness characteristics), processing quality (head rice rate), and cooking and eating quality (indicators such as amylose content and gel consistency). All determinations were set with three replicates to ensure data reliability.

### 4.7. Evaluation of Eating Quality Traits

After harvest and drying, rice grains were stored for 30 days to balance moisture content. Brown rice was prepared using a huller, polished with a rice polisher, and sieved with a broken rice separator to obtain intact milled rice. Rice flour was collected by grinding milled rice with a cyclone mill and passing it through a 100-mesh sieve. The rice quality was evaluated according to the industry standard (NY/T595-2013). The chalkiness rate and length–width ratio were determined using an appearance quality analyzer. Taste, stickiness, and hardness were measured with a texture analyzer and a hardness/stickiness analyzer after rice cooking. The amylose content was determined using the enzyme-linked immunosorbent assay, and the gel consistency was determined according to the national standard (GB/T 22294-2008). The alkali spreading value was measured according to the industry standard (NY 147-88). Pasting properties were analyzed using a Brabender Micro Visco-Amylo-Graph, with a measurement range of 300 cm·g, a rotation speed of 250 rpm, and the unit expressed in millipascal-seconds (mPa·s). The operational procedure was as follows: heating from ambient temperature to 95 °C at a rate of 7.5 °C/min and holding for 5 min; cooling down to 50 °C at a rate of 7.5 °C/min and holding for 1 min at the final temperature.

### 4.8. Statistical Analysis

Statistical analyses were performed using GraphPad Prism 8 software. The data were analyzed by one-way ANOVA combined with Tukey’s test. Significant differences (*p* ≤ 0.05) were indicated by different letters. All experiments were conducted with at least three biological replicates.

## Data Availability

All datasets generated for this study are included in the article/Supplementary file.

## Supplementary Materials

The following supporting information can be downloaded at: https://www.mdpi.com/article/10.3390/plants15020323/s1. Figure S1. Breeding process of hybrid rice cultivar Ruanfengyou 610 with dual resistance to rice blast and bacterial leaf blight. Figure S2. Evaluation of RB resistance in rice germplasm resources with different resistance alleles via the spray inoculation method. Figure S3. The resistance to BLB in rice germplasm resources with different Xa23 allele genotypes. Table S1. PARMS primer sets for genes related to resistance to blast and blight in rice. Table S2. Genotyping of targeted genes in 384 rice varieties. Table S3. Identification of RB resistance in Ruanfeng B and its parents. Table S4. Identification of RB resistance in Gui 610 and its parents. Table S5. Identification of BLB resistance in Gui 610 and its parents. Table S6. Identification of main agronomic traits from the 2 year regional trial.

## Abbreviations

The following abbreviations are used in this manuscript:

PARMS	Penta-primer Amplification Refractory Mutation System
SNP	Single Nucleotide Polymorphism
RB	Rice Blast
BLB	Bacterial Leaf Blight
TALE	Transcription Activator–Like Effectors
EBE	Effector Binding Element
AvrXa23	Avirulence protein for Xa23 resistance gene
CTAB	Cetyltrimethylammonium Bromide
TE	Tris-EDTA
dNTP	deoxy-ribonucleoside triphosphate
ROX	6-Carboxy-X-Rhodamine
HEX	Hexachloro-6-carboxyfluorescein
FAM	5 (6)-Carboxyfluorescein
