# Supplementary material for: Pyramiding Pita, Pigm, Pi2, and Xa23 to Develop Hybrid Rice with Dual Resistance to Rice Blast and Bacterial Blight
Source: Plants (Basel). 2026 Jan 21;15(2):323. doi: 10.3390/plants15020323 (PMC12845295; doi:10.3390/plants15020323)
Supplement: Supplementary file 1 [file plants-15-00323-s001.zip › Supplementary Figure.pdf]

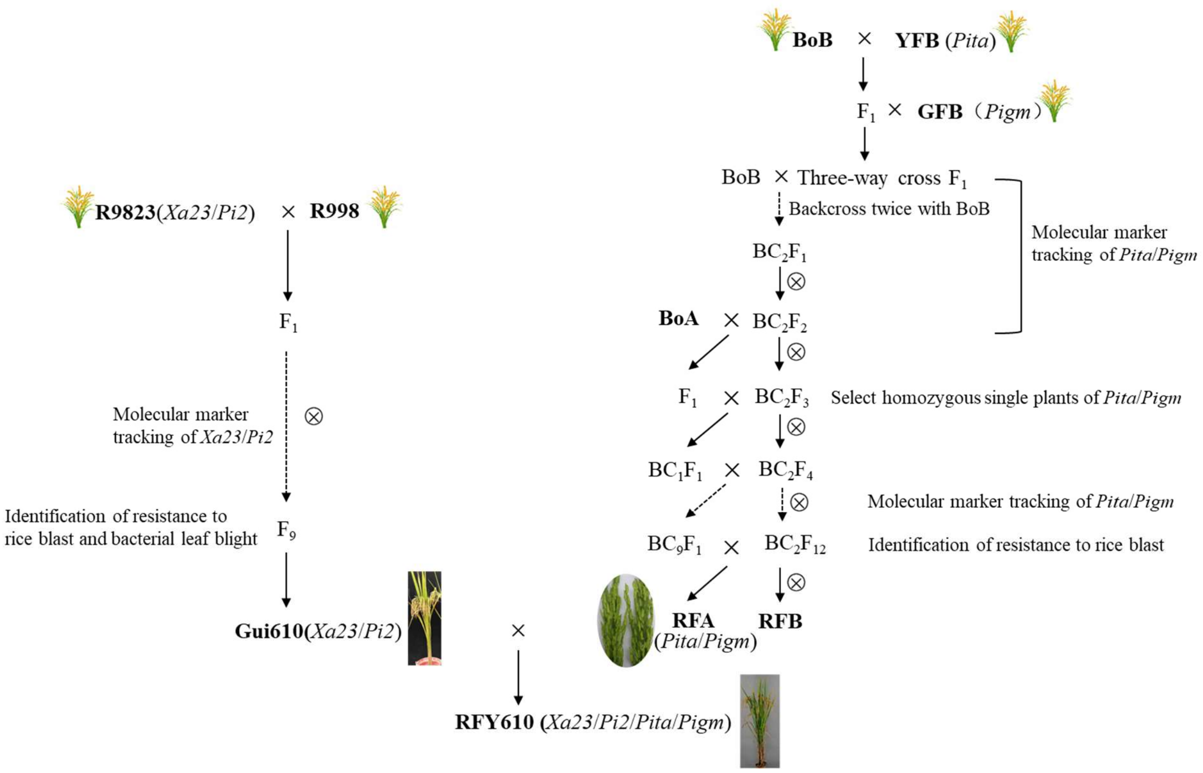

Figure S1. Breeding process of hybrid rice cultivar Ruanfengyou 610 with dual resistance to rice blast and bacterial leaf blight. YFB: Yuanfeng B; GFB: GufengB; RFB: Ruanfeng B; RFA: Ruanfeng A; RFY610: Ruanfengyou 610.

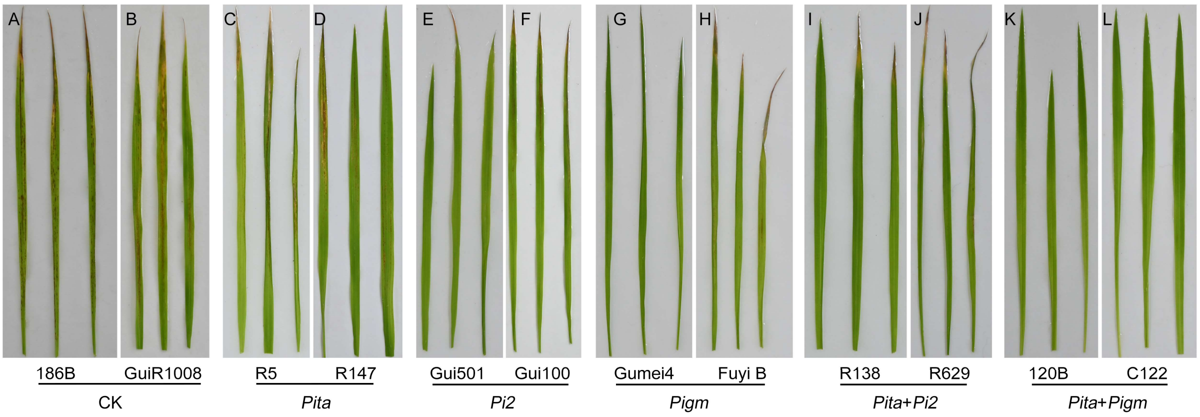

Figure S2. Evaluation of RB resistance in rice germplasm resources with different resistance alleles via the spray inoculation method. (A-B) Rice germplasm resources harbored the susceptible alleles *pita*, *pi2*, *pigm*. (C-L) The annotation at the bottom of the image indicates the resistance allele genotypes and combinations of rice germplasm resources.

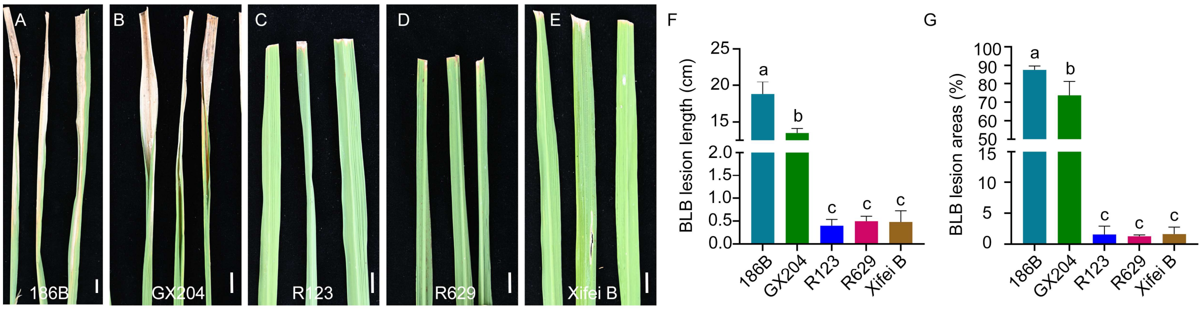

Figure S3. The resistance to BLB in rice germplasm resources with different *Xa23* allele genotypes. (A-B) Rice germplasm resources carrying the susceptible gene *xa23*. (C-E) Rice germplasm resources carrying the disease-resistant gene *Xa23*. (F) Statistical results of lesion length of BLB. (G) Statistical results of BLB lesion areas.
